# Unexpected toxicity of CDK4/6 inhibitor palbociclib and radiotherapy

**DOI:** 10.1002/cnr2.1470

**Published:** 2021-06-18

**Authors:** Evert S. M. van Aken, Aart Beeker, Ilse Houtenbos, Floris J. Pos, Sabine C. Linn, Paula H. M. Elkhuizen, Monique C. de Jong

**Affiliations:** ^1^ Department of Radiation Oncology Netherlands Cancer Institute – Antoni van Leeuwenhoek Amsterdam The Netherlands; ^2^ Department of Medical Oncology Spaarne Gasthuis Hoofddorp The Netherlands; ^3^ Department of Medical Oncology Netherlands Cancer Institute – Antoni van Leeuwenhoek Amsterdam The Netherlands

**Keywords:** breast cancer, clinical observations, radiation therapy, targeted therapy

## Abstract

**Background:**

Cyclin‐dependent kinase (CDK) 4/6 inhibitors have recently been approved for the treatment of hormone receptor–positive and HER2‐negative metastatic breast cancer in association with endocrine therapy in postmenopausal women. Data on the interaction of CDK4/6 inhibition and radiotherapy are scarce, but some studies show unexpected toxicity.

**Cases:**

We report three cases of unexpected severe or prolonged soft tissue, skin, and gastrointestinal toxicity in patients treated with a combination of radiotherapy and the CDK4/6 inhibitor palbociclib.

**Conclusion:**

These cases indicate a possible interaction between radiotherapy and palbociclib. Therefore, we recommend using radiotherapy cautiously when combined with CDK4/6 inhibitors.

## 1 BACKGROUND

Cyclin‐dependent kinase (CDK) 4/6 inhibitors like abemaciclib, ribociclib, and palbociclib have recently been approved for the treatment of hormone receptor–positive and HER2‐negative metastatic breast cancer in combination with endocrine therapy in postmenopausal women.^1^, ^2^, ^3^ Many of these women are candidates for radiotherapy during palbociclib use due to (oligo)progression or local complaints.^4^ However, data on the interaction between CDK4/6 inhibition and radiotherapy are scarce.

Several small series of patients receiving radiotherapy during treatment with a CDK4/6 inhibitor have been published. Most of these series do not report unexpected toxicity for the combination of CDK4/6 inhibitors and radiotherapy.^5^, ^6^, ^7^, ^8^, ^9^, ^10^, ^11^, ^12^

Nevertheless, grade 3 (G3) skin and gastrointestinal toxicity are reported for the combination of radiotherapy and CDK4/6 inhibitors. Two case reports describe a G3 colitis after 10 × 3 Gy while on palbociclib.^13^, ^14^ Two retrospective studies show that the combination is generally safe, but the authors also report a G3 ileitis in one patient (10 × 3 Gy) and G3 skin toxicity in two patients (radiation dose not mentioned).^15^, ^16^ Grade 3 esophagitis is described in two case reports (30 × 2 Gy and 5 × 4 Gy), combined with G3 dermatitis in one patient.^17^, ^18^ Also, a conference abstract reports unexpected pronounced pulmonary fibrosis and radiation pneumonitis.^19^

We present three patients with unexpected toxicity after radiotherapy in combination with palbociclib (Table 1), which indicates a possible severe interaction between these treatments.

**TABLE 1:** Patient characteristics

	Patient 1	Patient 2	Patient 3
Age	64	60	58
Gender	Female	Female	Female
Relevant comorbidities	None	None	None
Oncological history (time prior to the radiotherapy courses described in case report)	12 years: pT1N1M0 breast cancer (right breast), ER+, PR−, HER2−. Breast conserving surgery with sentinel node procedure, followed by axillary lymph node dissection. Adjuvant radiotherapy, chemotherapy, and hormonal therapy. 6 years: Stop hormonal therapy. 2 months: Bone metastases: start with palbociclib and letrozole.	5 years: pT1N0Mx breast cancer (left breast), ER+, PR+, HER2−. Breast conserving surgery with sentinel node procedure. Adjuvant radiotherapy, chemotherapy, and hormonal therapy. 2 months: Bone metastases: start with palbociclib and fulvestrant. Radiotherapy: 1 × 8 Gy, left hip.	19 years: T1N1 breast cancer (left breast), ER−, PR−, HER2−. Incomplete diagnostic excision, chemotherapy, second incomplete excision, third excision, and axillary lymph node dissection. Adjuvant radiotherapy and chemotherapy. 7 years: Breast cancer (left breast), ER+, PR+, HER2−. Salvage mastectomy with latissimus dorsi flap reconstruction. Adjuvant chemotherapy and hormonal therapy. 5 years: Breast cancer (left breast), ER+, PR+, HER2−. Induction chemotherapy and excision. Reirradiation to left thoracic wall and parasternal lymph nodes, combined with hyperthermia. Adjuvant hormonal therapy. 1 year: Metastasized breast cancer. Start with palbociclib and fulvestrant.
Radiation dose and target area	5 × 4 Gy, right pelvis	2 × 8 Gy, left hip (after 1 × 8 Gy)	17 × 3 Gy, mediastinum and right hilum
Palbociclib timing	Concurrent	Concurrent	Stopped 3–4 days before radiotherapy. Restarted 8–9 days after radiotherapy.
Observed toxicity	G3 enterocolitis, G3 diarrhea	G3 edema, G2 pain, G2 dermatitis	Prolonged G2 dysphagia with G2 esophageal ulcer

Abbreviations: ER, estrogen receptor; G, grade; PR, progesterone receptor.

## 2 CASES

### 2.1 Patient 1

**FIGURE 1:**
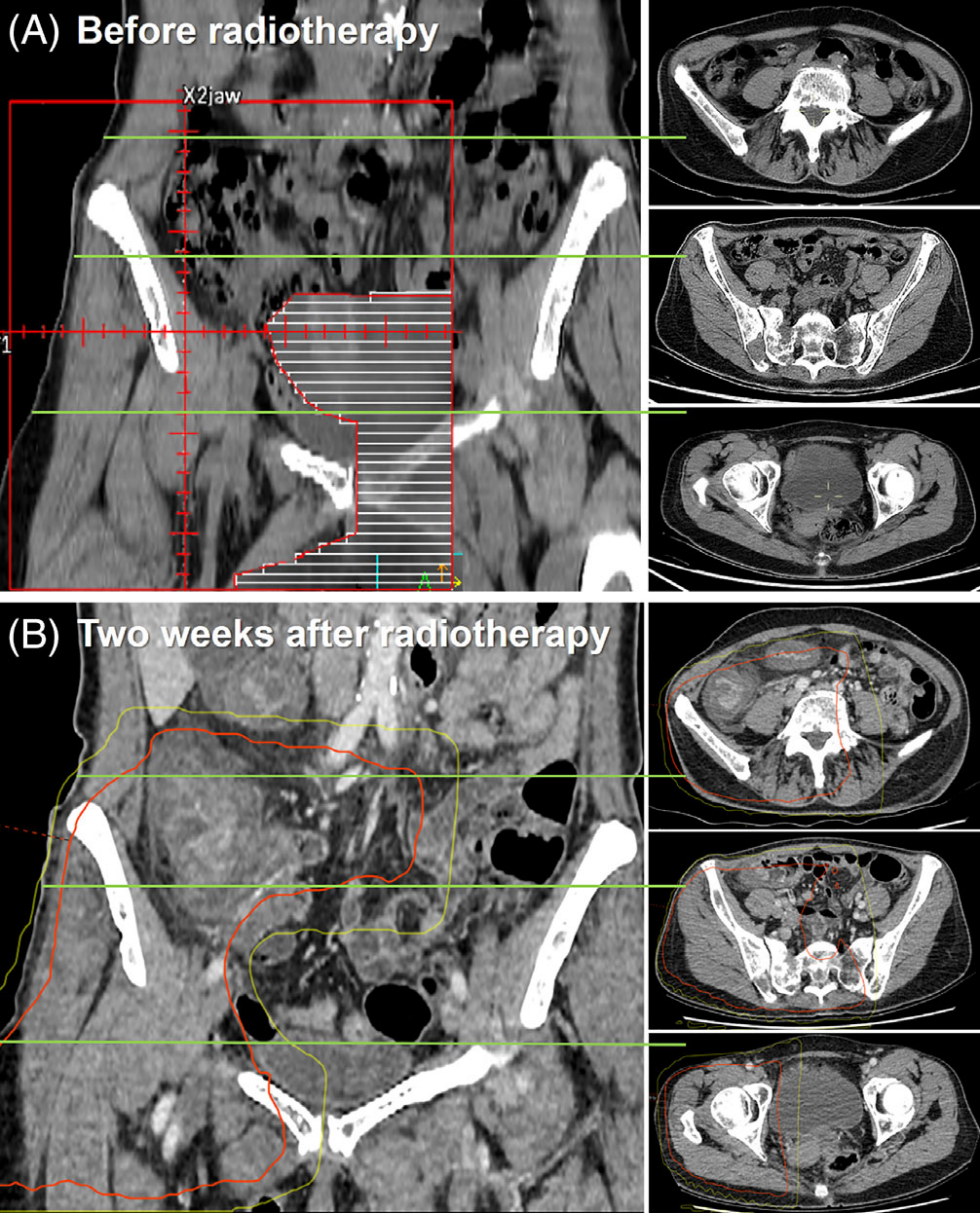
Radiotherapy field set‐up (A) and the coronal and corresponding axial diagnostic contrast‐enhanced abdominal CT 2 weeks after radiotherapy with an overlay of the 10 Gy (yellow) and 20 Gy (red) isodose lines (B). CT, computed tomography

A 64‐year‐old woman with recently diagnosed bone metastases of a previously treated breast cancer was treated with letrozole and palbociclib. Two months after the start of systemic treatment, palliative radiotherapy (20 Gy in 5 fractions, once daily) was prescribed to symptomatic bone metastases in the right pelvis, using two opposing 10 MV fields. An EQD_2,α/β = 10 Gy_ (equivalent dose in 2‐Gy fractions) of 23 Gy was delivered to bowel loops inside the field. Palbociclib was continued during radiotherapy. Days after the last fraction, our patient experienced a severe enterocolitis, for which she was hospitalized for 10 days. On a diagnostic computed tomography (CT), bowel loops inside the radiotherapy field were clearly swollen (Figure 1). Treatment consisted of analgesics, antibiotics, and nil per os. Two months after radiotherapy, she was still not fully recovered, with occasional diarrhea and use of fortified drinks.

### 2.2 Patient 2

**FIGURE 2:**
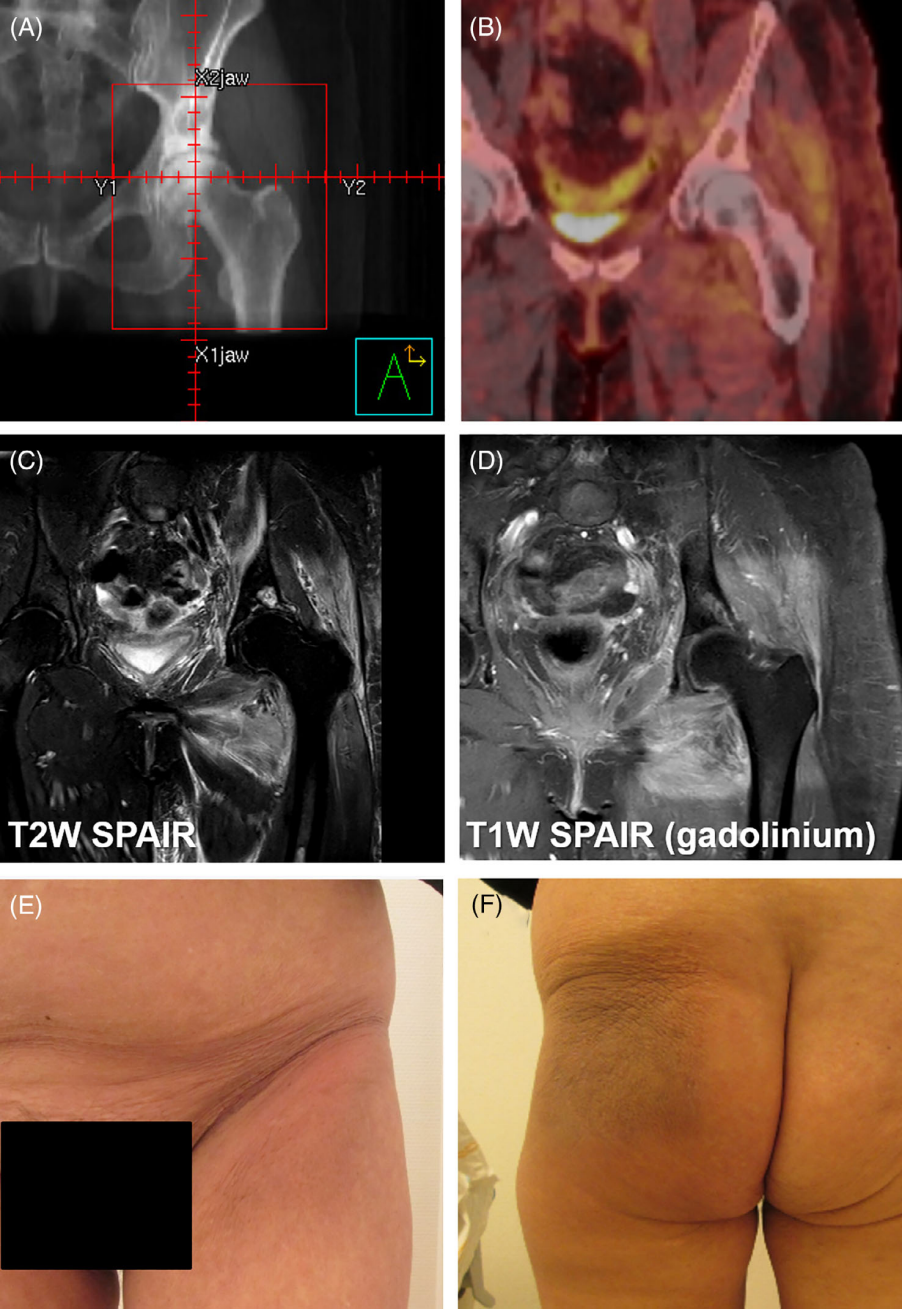
Radiotherapy field set‐up (A), FDG PET/CT 4 months after radiotherapy (B), MRI 5 months after radiotherapy (C, D), and clinical images 5 months after radiotherapy (E, F). W, weighted, SPAIR, spectral‐attenuated inversion recovery, FDG PET/CT, fluorodeoxyglucose positron emission tomography combined with computed tomography

A 60‐year‐old woman with metastatic breast cancer was treated with fulvestrant and palbociclib. At the start of her systemic treatment, she received palliative radiotherapy (8 Gy in a single fraction, field shown in Figure 2A) to a painful bone metastasis in the left hip, resulting in a short‐term reduction of pain. Hoping to increase effectiveness, she received a second course of radiotherapy 2 months later (16 Gy in two fractions). Radiotherapy was administered using two opposing fields (10 MV), resulting in a total dose just below the skin of EQD_2,α/β = 3 Gy_ 32 Gy, increasing to 53 Gy around the femur.

Four months after the last radiotherapy, she experienced edema, redness, and pain of her left upper leg/hip. A deep venous thrombosis was excluded, and treatment with antibiotics had no effect. Clinical photographs taken 5 months after the completion of the second radiotherapy course show clear evidence of skin discoloration and induration within the radiation field (Figure 2E,F). An FDG PET/CT (fluorodeoxyglucose positron emission tomography combined with computed tomography) showed no evidence of disease progression, but a moderate generalized uptake of FDG around the left hip (Figure 2B). Various MRI (magnetic resonance imaging) sequences showed increased signal intensity in the area of the original radiotherapy field, most pronounced on the gadolinium‐enhanced T1‐weighted SPAIR (spectral‐attenuated inversion recovery) MRI (Figure 2C,D). Six months after radiotherapy, she was using an equivalent of more than 250 mg oral morphine per 24 h and 20 mg of prednisone, resulting in some reduction of swelling but no satisfactory pain relief.

### 2.3 Patient 3

**FIGURE 3:**
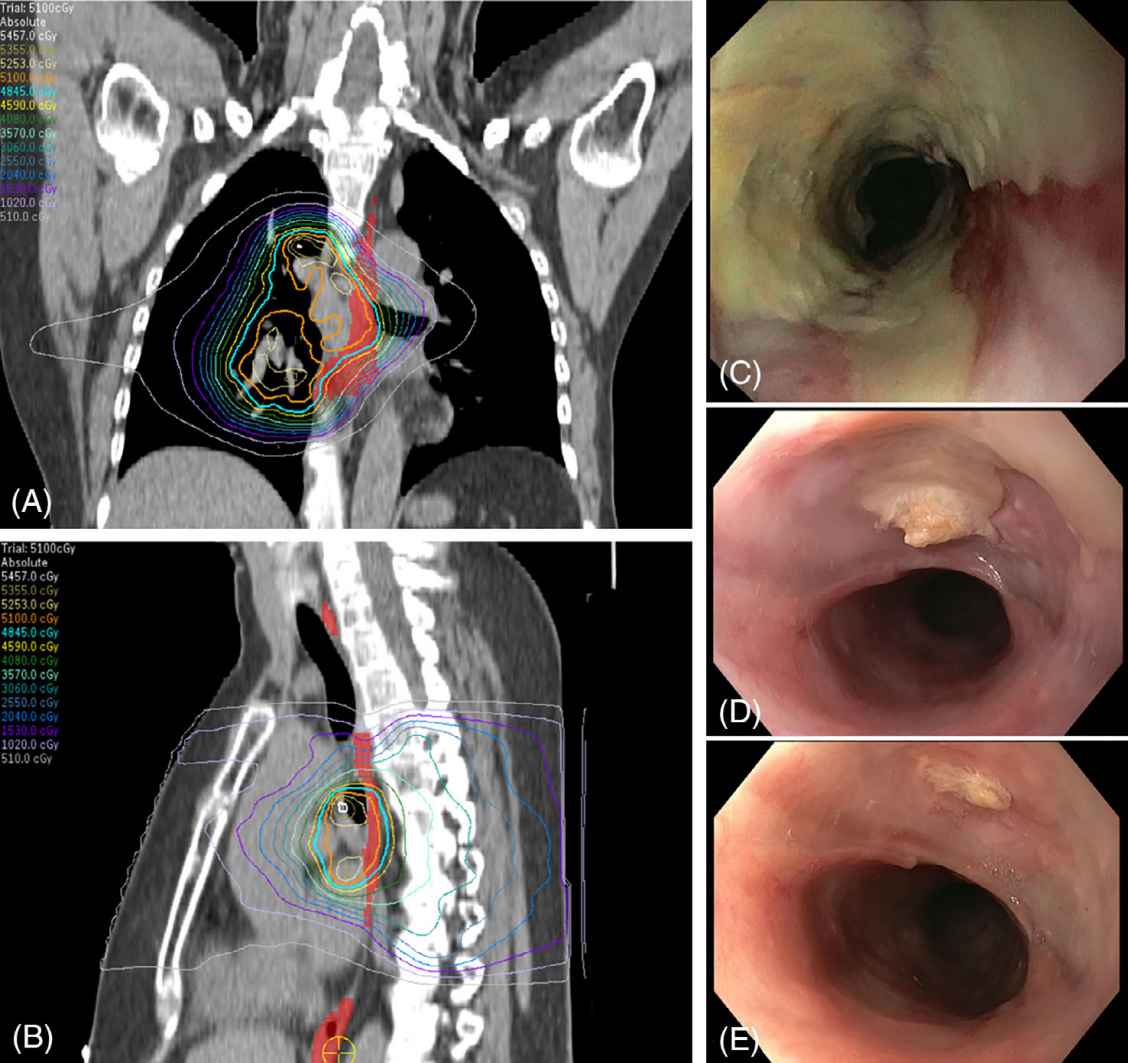
Radiotherapy isodose lines (A, B) with 51 Gy (orange) isodose line and esophagus (red area). Images from the first (C), second (D), and third (E) esophagoscopy

A 58‐year‐old woman with metastatic breast cancer was treated with fulvestrant and palbociclib. In the past, she was treated with 50 Gy (tangential fields) + 15 Gy electron boost to the left breast. For this treatment, a substantial dose to the esophagus is very unlikely. Fourteen years later, she was treated with 46 Gy (fractions of 2 Gy) reirradiation combined with superficial hyperthermia to the left thoracic wall and parasternal lymph nodes because of tumor recurrence. The estimated EQD_2,α/β = 10 Gy,_ to the mid‐thoracic esophagus was 21 Gy. Because of hilar and mediastinal oligoprogression^4^ during fulvestrant and palbociclib 5 years later, she received radiotherapy (17 × 3 Gy, once daily, field shown in Figure 3A,B) to the mediastinum and right hilum, using volumetric modulated arc therapy (VMAT). The V50_α/β = 10 Gy_ (relative volume receiving an EQD_2,α/β = 10 Gy_ of at least 50 Gy) was 13.3%, correlating with a G ≥ 2 esophagitis risk of around 25%.^20^ Palbociclib was stopped 3–4 days before radiotherapy and restarted 8–9 days after the last fraction. During the radiotherapy treatment, she developed progressive dysphagia with an inadequate response to analgesics and sucralfate. After an initial slight improvement at 2 weeks after radiotherapy, symptoms worsened and did not improve with pantoprazole. An esophagoscopy was performed 3 months after the last radiotherapy, showing an ulcer with a pinpoint stenosis (Figure 3C). The location of the ulcer corresponded with the high‐dose region of the last radiotherapy course.^21^ A biopsy was taken and showed squamous epithelium with some inflammation and neither dysplasia nor carcinoma. Palbociclib was then stopped, which resulted in a gradual improvement of her symptoms. A second esophagoscopy was performed 1.5 months later, which only showed a small, superficial ulcer and a decrease of the stenosis (Figure 3D). Palbociclib was restarted 5.5 months after the last radiotherapy, following a third esophagoscopy, which showed visible improvement (Figure 3E). Palbociclib was continued for five more months until disease progression, without new worsening of her symptoms.

## 3 DISCUSSION

We present three cases of unexpected severe or prolonged soft tissue, skin, and gastrointestinal toxicity that occurred in patients receiving palbociclib and radiotherapy. We did not discover alternative explanations for the observed toxicities. Dose delivery during treatment was verified using electronic portal image dosimetry and was delivered as planned. Side effects were only present inside the radiotherapy field and are extremely uncommon for equivalent doses to bowel and soft tissue/skin in the first two cases. In the third case, dysphagia was not unexpected (especially not after previous radiotherapy), but the recovery pattern was unusual. An initial recovery was followed by worsening after restart of palbociclib, and improvement was seen only after discontinuation of palbociclib. This emphasizes the possible negative role of palbociclib during repopulation of healthy tissues after radiotherapy.

A pre‐existing increased intrinsic sensitivity to ionizing radiation is highly unlikely in these patients, since they had radiotherapy for their primary breast cancer without serious unexpected toxicity, and there were no known risk factors for increased radiotherapy toxicity. Additionally, there was no reason to assume different pharmacodynamics in these patients.

It is worth noting that the patients in this case series received hypofractionated radiotherapy, which may lead to a less beneficial therapeutic ratio with regard to normal tissue complications, but this is particularly the case for late‐responding tissues.^22^, ^23^ Furthermore, hypofractionated radiotherapy is often applied as a convenient and effective therapy for palliative treatment indications.^22^

In the introduction, we summarized the literature regarding the combination of radiotherapy with CDK4/6 inhibitors. The observations in our case series are in line with previous reports showing several cases of G3 skin and gastrointestinal toxicity.^13^, ^14^, ^15^, ^16^, ^17^, ^18^ However, the nature and limited size of these studies, including our study, make it difficult to reliably predict the absolute risk of increased toxicity.

### 3.1 Possible (radio)biological mechanism

Palbociclib is a CDK4/6 inhibitor that inhibits cell division by causing a G1/S block.^24^ While this is advantageous for the elimination of fast‐dividing cancer cells, cell division is pivotal to repair/repopulate normal tissues after radiotherapy. Inhibition could therefore lead to increased and prolonged normal tissue damage. Although the main mechanism of palbociclib is cell cycle inhibition, the possibility of radiosensitization by an off‐target effect cannot be excluded. A report from Huang et al. describes radiosensitization by mediating the DNA damage response.^25^

## 4 CONCLUSION

We present two cases suggesting severe unexpected normal tissue radiosensitization by palbociclib and one case with delayed recovery from acute normal tissue toxicity by palbociclib. We recommend using radiotherapy cautiously when combined with CDK4/6 inhibitors. If palbociclib is discontinued during radiotherapy, withholding palbociclib until recovery from acute radiotherapy toxicity appears sensible. As the number of women on CDK4/6 inhibitors increases, both medical and radiation oncologists should be aware of a possible interaction when patients are referred for radiotherapy.

## CONFLICT OF INTEREST

S.L. is an advisory board member for AstraZeneca, Cergentis, IBM, Pfizer and Roche and received grants paid to the institute from Agendia, AstraZeneca, Eurocept‐pharmaceuticals, Genentech, Novartis, Pfizer, Roche, Tesaro, and Immunomedics outside of this study. In addition, S.L. received institutional nonfinancial support from Agendia, AstraZeneca, Bayer, Daiichi‐Sankyo, Genentech, IBM, Immunomedics, Novartis, Pfizer, Roche, and Tesaro outside of this study. The other authors declare no conflicts of interest.

## AUTHOR CONTRIBUTIONS

All authors had full access to the data in the study and take responsibility for the integrity of the data and the accuracy of the data analysis. *Conceptualization*, A.B., I.H., F.P., S.L., P.E., M.J.; *Data Curation*, E.A., M.J.; *Visualization*, E.A., M.J.; *Writing‐Original Draft*, E.A., M.J.; *Writing‐Review & Editing*, E.A., A.B., I.H., F.P., S.L., P.E., M.J.

## ETHICAL STATEMENT

All patients agreed to anonymized publication of their data. As this is a case series, no institutional approval was required. All authors contributed to the collection of the data, the analysis of the data, and the writing of this manuscript. All authors had full access to the data in the study and take responsibility for the integrity of the data and the accuracy of the data analysis.

## Data Availability

Data sharing not applicable to this article as no datasets were generated or analyzed during the current study.
